# Effects of radiation therapy on the meibomian glands and dry eye in patients with ocular adnexal mucosa-associated lymphoid tissue lymphoma

**DOI:** 10.1186/s12886-019-1301-0

**Published:** 2020-01-13

**Authors:** Sung Eun Kim, Hee Jung Yang, Suk-Woo Yang

**Affiliations:** Department of Ophthalmology and Visual Science, Seoul St. Mary’s Hospital, College of Medicine, The Catholic University of Korea, 222 Banpo-daero, Seocho-gu, Seoul, 06591 South Korea

**Keywords:** Meibomian gland, Radiation therapy, Ocular adnexal mucosa-associated lymphoid tissue lymphoma, MALT lymphoma, Dry eye

## Abstract

**Background:**

Radiation therapy (RT) is the treatment of choice in patients with low-grade ocular adenexal mucosa-associated lymphoid tissue lymphoma (OAML) and many of them experience post-RT dry eye with varying severity. The purpose of the present study was to investigate ocular effects of RT on meibomian glands and dry eye by directly visualizing structural changes. Secondly, we focused on the comparison of two groups of patients according to tumor location and radiation technique.

**Methods:**

Sixty-four eyes with OAML of conjunctiva, orbit, lacrimal gland, or lacrimal sac were grouped into conjunctival lymphoma and “orbital-type” lymphoma (i.e., orbit, lacrimal gland, and lacrimal sac). Subjects were investigated for morphological changes in meibomian glands by meiboscore grading system. Radiation technique was examined and Ocular Surface Disease Index (OSDI) questionnaire, Schirmer’s test, tear film break-up time (TBUT), slit lamp examination of corneal surface and lid margin abnormality were conducted before and after RT.

**Results:**

The increase in meiboscore was statistically significant over time after RT in both groups (*P* < 0.001). The extent of increase in meiboscore was significantly greater in the “orbital-type” lymphoma group than in the conjunctival lymphoma group (*P* < 0.001). The changes in OSDI, TBUT, corneal fluorescein staining score and lid margin abnormality score after RT were significantly different across two groups (*P* = 0.042, 0.001, 0.035 and 0.001, respectively). Schirmer’s value decreased after RT in both groups. Dry eye symptoms were most severe right after RT in both groups, but a gradual resolution was noted in most patients with conjunctival lymphoma, whereas symptoms persisted in “orbital-type” lymphoma patients. The OSDI score and corneal fluorescein staining score were positively correlated with meiboscore in “orbital-type” patients at post-RT 6 months (*r* = 0.43, *P* = 0.04; *r* = 0.39, *P* = 0.03, respectively).

**Conclusions:**

Patients with OAML had different degrees of morphological changes in meibomian glands according to tumor location and radiation technique. “Orbital-type” lymphoma patients are more likely to experience severe injury to meibomian glands, which eventually leads to persistent dry eye. Patients with “orbital-type” lymphoma should be well informed of post-RT damage on meibomian glands and persistent dry eye.

## Background

The most common subtype of primary ocular adnexal lymphoma is marginal zone lymphoma of mucosa-associated lymphoid tissue (MALT) [1]. Radiation therapy is the treatment of choice for low-grade primary ocular adnexal MALT lymphoma (OAML) as it is very effective in terms of local control and generally well-tolerated [2–5]. However, toxicity following irradiation is inevitable and results in adverse effects to the ocular structures [6, 7]. Previous studies have reported serious ocular morbidities that can occur after radiation therapy (RT) [8, 9]. However, because low-dose radiation is predominantly the treatment of choice, complications such as cataract, retinitis, and optic neuropathy are rarely encountered [10–13]. Above all, one of the most common adverse effects after RT is dry eye syndrome. Concerning symptoms of ocular dryness can occur during a course of fractionated radiotherapy [12] and a progression of dry eye is commonly experienced by patients. Meibomian gland dysfunction (MGD) is one of the most common causes of dry eye syndrome [14, 15] and, to our knowledge, there has been no study aimed at directly visualizing structural changes in the meibomian glands sequentially over time beginning with the initiation of RT. In the current study, meibomian gland morphology was sequentially examined at each visit during the post-radiation period for 6 months and compared with that of the pre-radiation meibomian glands. Additionally, we focused on the comparison of two groups of patients (those with conjunctival lymphoma and those with “orbital-type” lymphoma) because the radiation technique used was different depending on the location of the tumor [16–18].

## Methods

A prospective study was performed from March 2017 to December 2018. The institutional review board of the Catholic University of Korea approved the research protocol and the study was conducted in accordance with the tenets of the Declaration of Helsinki. Sixty-four patients were identified as having presented with OAML of the conjunctiva, orbit, lacrimal gland, and lacrimal sac. Patients in whom external beam radiation therapy (EBRT) was selected as a primary treatment were included in the study. Patients who had received RT or chemotherapy previously or those who had severe dry eye, conjunctival or corneal disease, a history of contact lens use, medications such as glaucoma medications or other systemic diseases such as androgen insensitivity syndrome, and Sjogren’s syndrome that could have an influence on the meibomian gland were excluded. The patients underwent at least 6 months of follow-up after completion of RT. All ophthalmologic examinations including meibography were carried out by a single examiner (SK). Patients with post-RT dry eye syndrome were treated with artificial tears, ointments and anti-inflammatory agents or topical steroids according to the severity of the condition. They were encouraged to take omega-3 fatty acid dietary supplementation and carry out warm compresses.

### Radiation technique

Radiation records were reviewed with respect to radiation dose, radiation energy, and direction of the radiation beam (gantry angle) in each subject. The patients with OAML in the orbit, lacrimal gland, and lacrimal sac were newly grouped as “orbital-type” lymphoma and compared with patients with conjunctival lymphoma because the radiation protocol could be largely differentiated between these two groups according to the primary site of OAML. Therefore, we expected the influence of radiotherapy on the meibomian glands to vary between the two groups according to the different radiation protocols used.

### Meibography analysis

The non-contact infrared meibography system used in this study was described by Hwang et al. [19] The pictures of glands in the upper and lower eyelids were obtained and analyzed by a single ophthalmologist (SK) at each visit. The morphological changes were analyzed using the following “meiboscore” grading system introduced by Arita et al. [20]: grade 0, no loss of gland; grade 1, gland loss of < 33%; grade 2, gland loss of between 33 and 67%; and grade 3, gland loss of > 67% of the total area.

### Ophthalmologic examinations

Patients were examined before and after one, three, and 6 months of RT. At each visit, visual acuity, Schirmer’s test, the Ocular Surface Disease Index (OSDI) questionnaire [21], tear film break-up time (TBUT) test, slit lamp biomicroscopic examination of the corneal staining by Oxford grading scheme from 0 to 5 under cobalt blue illumination and lid margin abnormality were conducted in order. The post-radiation change in parameters was compared between the two groups at each visit and correlation of meiboscore with the ocular surface parameters was analyzed at the last follow-up.

### Ocular surface disease index

The OSDI questionnaire is comprised of 12 questions, with each question graded on a scale of 0 to 4. The total OSDI score was calculated on the basis of the formula: OSDI = [(sum of scores for all questions answered) × 100]/(total number of questions answered) × 4]. Thus, the OSDI was scored on a scale of 0 to 100, with higher scores representing greater disability.

### Lid margin abnormality

Lid margin abnormality was scored from 0 to 4 depending on the presence or absence of the following abnormalities: irregular lid margin, vascular engorgement, plugging of the meibomian gland orifices, and anterior or posterior replacement of the mucocutaneous junction [20].

### Statistical analysis

Statistical analysis was performed using the IBM SPSS Statistics for Windows, version 20.0 (IBM Corp., Armonk, N.Y., USA). A comparison of the mean change in meiboscore over time after RT between the conjunctival lymphoma group and the “orbital-type” lymphoma group was done using repeated measures analysis of variance (ANOVA). The mean change in the value of each ocular parameter before and after RT was compared using Wilcoxon signed rank test and between the two groups using Mann Whitney U-test. The correlation of meiboscore with the ocular surface parameters was analyzed by Spearman’s correlation test. Statistical significance was defined when the *P* value was < 0.05.

## Results

A total of 64 eyes of 64 patients, of whom 44 eyes had conjunctival lymphoma and 20 eyes had “orbital-type” (i.e., orbit, lacrimal gland, and lacrimal sac) lymphoma were included in this study from March 2017 to December 2018.The demographics of patients and the radiation techniques used are shown in Table 1. The median age at diagnosis was 41 years (range: 21 years to 79 years; two patients were older than 70 years), with a female predominance (males/females: 25/39). The mean age of those with “orbital-type” lymphoma was older than that of conjunctival lymphoma (54.9 years and 43.2 years, respectively, *P* = 0.147).

**Table 1:** Demographic data for the patients and the values for their radiation parameters

Primary site	Conjunctiva (*n* = 44)	Orbit (*n* = 11)	Lacrimal gland (*n* = 6)	Lacrimal sac (*n* = 3)
Age (years)	43.2 ± 15.9	60.4 ± 12.5	46.5 ± 16.5	51.3 ± 17.2
Gender (M:F)	16:28	7:4	1:5	1:2
Total radiation dose (cGy)	2340 – 2520	3060 – 3600	3060 – 3600	3060
Radiation energy	6–8 MeV (electron beam)	12–15 MeV (electron beam) or 6–10 MV (X-ray)	6 MV (X-ray)	6–10 MV (X-ray)
Underlying disease				
Diabetes Mellitus	3 (0.07%)	1 (0.09%)	0 (0%)	0 (0%)
Hypertension	5 (0.11%)	1 (0.09%)	1 (0.16%)	0 (0%)

Data are expressed as mean ± standard deviation or number

The total radiation dose and energy used were different among the two groups. In the conjunctival lymphoma group, megaelectron-volt (MeV) electron beams were used, whereas mega-volt (MV) X-rays or higher MeV was used in cases of “orbital-type” lymphoma. Conjunctival lymphomas were treated with an energy amount of 6 to 8 MeV and “orbital-type” lymphomas were treated with an energy amount of 12 to 15 MeV or 6 to 10 MV (X-ray).

The total radiation dose did not exceed 4000 centigray (cGy) in either group. Patients with conjunctival lymphoma received 2340 to 2520 cGy in 13 to 14 fractions over a period of about 3 weeks and those with “orbital-type” lymphoma received 3060 to 3600 cGy in 17 to 20 fractions over 4 weeks. A single anteroposterior field was used in all patients with conjunctival lymphoma, while various angles were individualized in patients with “orbital-type” lymphoma according to tumor size and location. Among 20 eyes of “orbital-type” lymphoma, two eyes of lacrimal sac and one eye of lacrimal gland lymphoma, respectively, received intensity-modulated RT (IMRT) with the radiation field centered on the tumor only. The other eyes of “orbital-type” lymphoma (17 eyes) received conventional three-dimensional conformal RT (3D-RT), with the radiation beam covering the entire orbital socket.

**Fig. 1:**
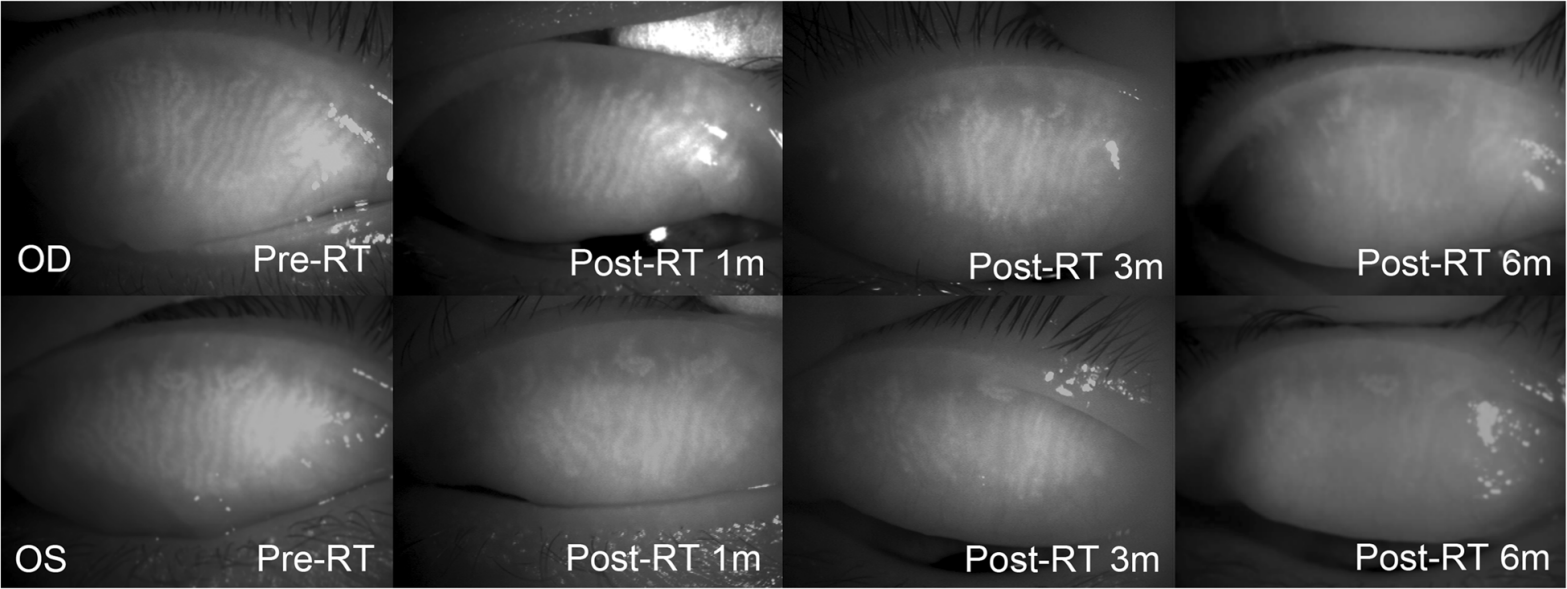
Post-radiation morphological changes in meibomian glands of a 38-year-old male. A total radiation dose of 2520 cGy and 10 MeV was delivered to conjunctival mucosa-associated lymphoid tissue lymphoma in both eyes

**Fig. 2:**
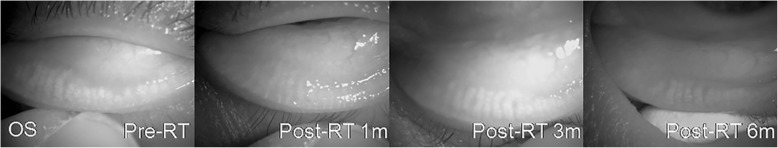
A 42-year-old female with conjunctival mucosa-associated lymphoid tissue lymphoma had a total radiation dose of 2520 cGy and 10 MeV in her left eye. Note the shortening of the meibomian glands

**Fig. 3:**
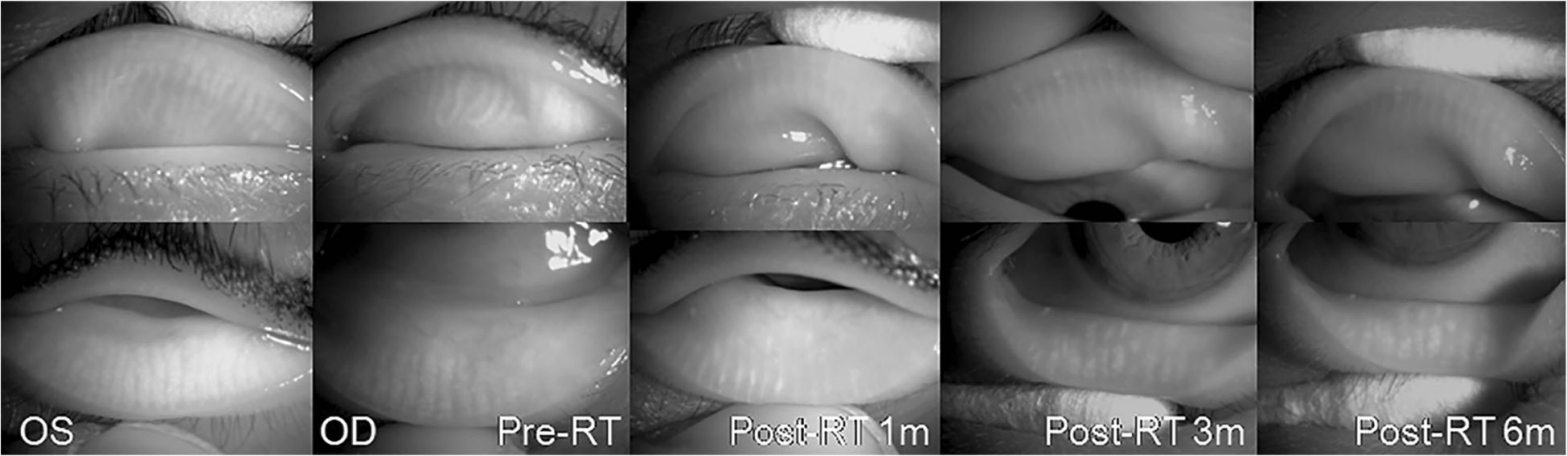
Comparison of the meibomian glands of irradiated eye with the fellow non-irradiated eye. A 54-year-old female patient with lacrimal gland mucosa-associated lymphoid tissue lymphoma in the right eye received a radiation dose of 3060 cGy and 6 MV X-ray. Note the distortion, shortening and dropout of meibomian glands in the irradiated eye

**Fig. 4:**
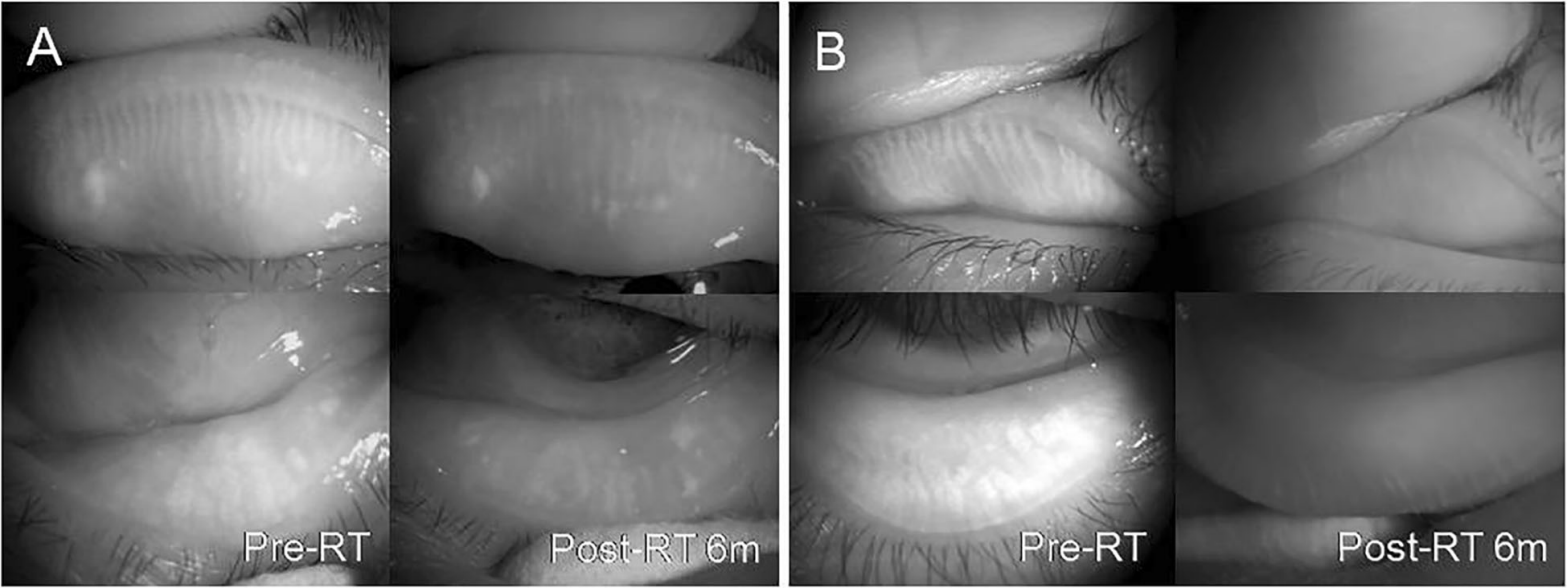
A 79-year-old female with orbital mucosa-associated lymphoid tissue lymphoma had a total radiation dose of 3060 cGy and 15 MeV in her left eye (**a**). A 41-year-old male with orbital mucosa-associated lymphoid tissue lymphoma had a total radiation dose of 3600 cGy and 12 MeV in his right eye (**b**). Note the slender ducts with invisible acinar. Distortion and loss of meibomian glands can be seen

In both groups, the comparison between the pre-radiation meibography and post-radiation meibography, and also with the non-treated fellow eye is described in Figs. 1, 2, 3 and 4. One patient with conjunctival MALT lymphoma had progressive changes in meibomian gland morphology during the 6 months of the follow-up period, as shown in Fig. 1 and the other patient experienced shortening of meibomian glands after RT, as shown in Fig. 2. The presence of morphological damage could also be demonstrated in the irradiated eyes as compared with in the normal eyes, and such case is shown in Fig. 3. Figure 4 shows the most severe cases with significant damage in the meibomian glands.

In the conjunctival lymphoma group, 11 eyes (25%) experienced morphological changes and the average of the meiboscore increased from 0.1 ± 0.3 before RT to 0.4 ± 0.6 at 6 months post-RT in the upper eyelid and from 0.2 ± 0.5 before RT to 0.4 ± 0.7 at 6 months post-RT in the lower eyelid (*P* < 0.001). In the “orbital-type” lymphoma group, on the other hand, 12 eyes (60%) exhibited morphological changes. The increase in meiboscore was much greater than that in the conjunctival lymphoma group, as it increased from 0.3 ± 0.5 before RT to 1.7 ± 1.0 at 6 months post-RT in upper eyelid and from 0.0 ± 0.0 before RT to 1.3 ± 1.3 at 6 months post-RT in the lower eyelid (*P* < 0.001). The increase in meiboscore was statistically significant over time after RT (*P* < 0.001) and the extent of increase in meiboscore was significantly greater in the “orbital-type” lymphoma group than in the conjunctival lymphoma group (*P* < 0.001). (Table 2).

**Table 2:** Mean changes in meiboscore after radiation therapy (RT) according to the location of OAML

Primary site		Pre-RT	Post-RT 1 m	Post-RT 3 m	Post-RT 6 m	*P* value
Conjunctival (*n* = 44 eyes)	Upper	0.1 ± 0.3	0.3 ± 0.7	0.4 ± 0.5	0.4 ± 0.6	< 0.001*
	Lower	0.2 ± 0.5	0.4 ± 0.6	0.4 ± 0.7	0.4 ± 0.7	< 0.001*
Orbital-type (*n* = 20 eyes)	Upper	0.3 ± 0.5	1.3 ± 0.9	1.5 ± 0.8	1.7 ± 1.0	< 0.001*
	Lower	0.0 ± 0.0	1.2 ± 1.0	1.3 ± 1.3	1.3 ± 1.3	< 0.001*
*P* value*		< 0.001*	< 0.001*	< 0.001*	< 0.001*	

OAML: ocular adnexal mucosa-associated lymphoid tissue lymphoma; RT: radiation therapy *Values with statistical significance are indicated with an asterisk (*)

**Fig. 5:**
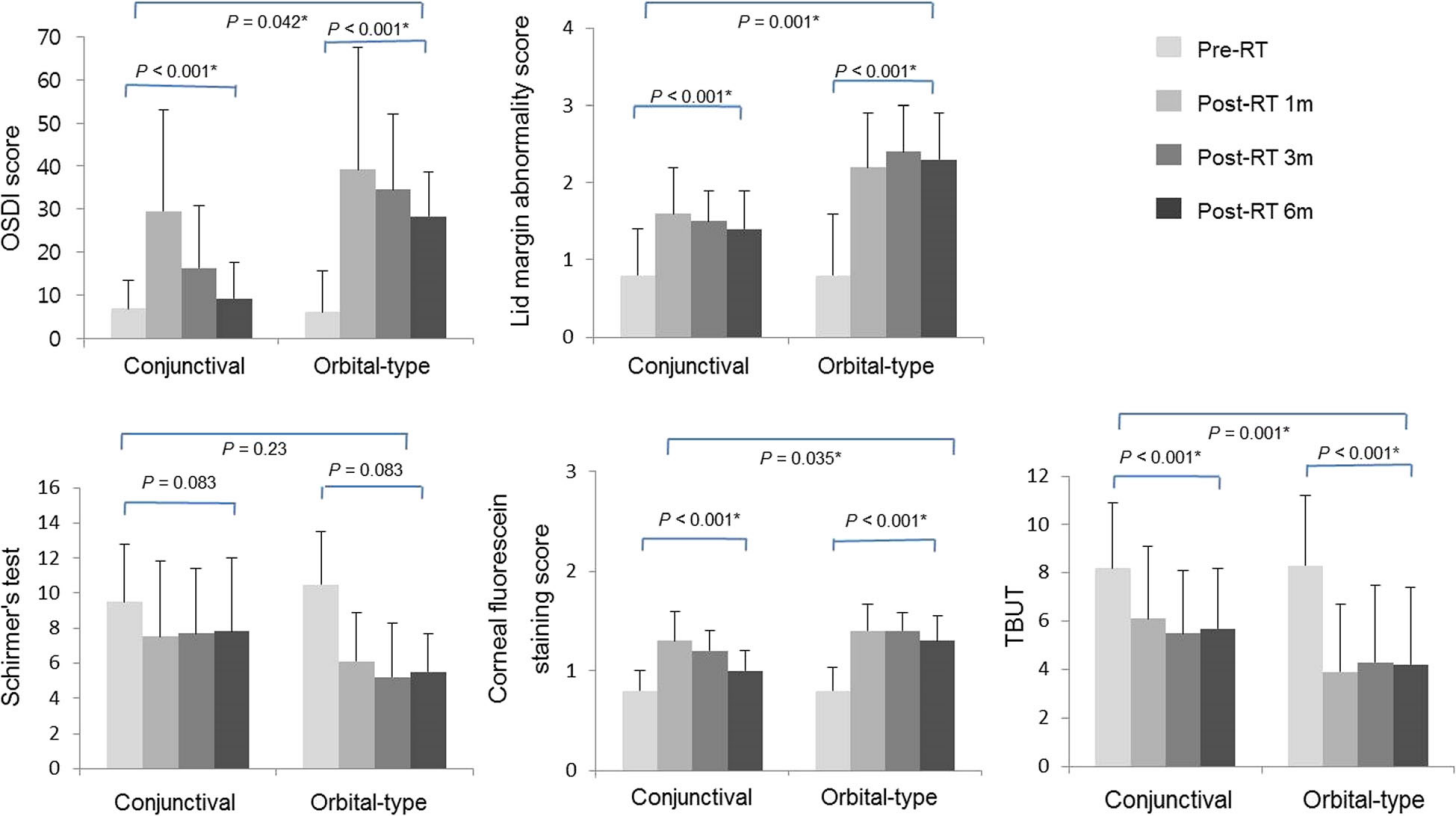
Comparison of ocular parameters in patients with ocular adnexal mucosa-associated lymphoid tissue lymphoma. Ocular parameters before radiation therapy (RT) and at 1, 3, and 6 months after RT were compared according to the location of OAML. Values with statistical significance are indicated with an asterisk (*). OSDI: ocular surface disease index; TBUT: tear film break-up time

The periodic changes and comparison of OSDI, Schirmer’s test, TBUT, corneal fluorescein staining score and lid margin abnormality score between the two groups are summarized in Fig. 5. In terms of the measured values, all parameters except Schirmer’s test demonstrated a statistically significant increase in impairment at 6 months post-radiation. The changes in OSDI, TBUT, corneal fluorescein staining score and lid margin abnormality score at 6 months after RT were significantly different across the two groups (*P* = 0.042, 0.001, 0.035 and 0.001, respectively). Notably, the increase in scores of OSDI, corneal fluorescein staining and lid margin abnormality and the decrease in TBUT were greater in patients with “orbital-type” lymphoma. Schirmer’s test value decreased after RT in both groups, but its difference was not found to be statistically significant between the groups. The correlation of meiboscore with ocular surface parameters is shown in Table 3. The OSDI score and corneal fluorescein staining score were positively correlated with meiboscore in “orbital-type” patients at post-RT 6 months (*r* = 0.43, *P* = 0.04; *r* = 0.39, *P* = 0.03, respectively).

**Table 3:** Correlation of meiboscore with ocular surface parameters in conjunctival and “orbital-type” OAML

	Conjunctival OAML	“Orbital-type” OAML
	Meiboscore (U + L)	Meiboscore (U + L)
OSDI	*r* = 0.38	*r* = 0.43
	*P* = 0.45	*P* = 0.04^a^
Schirmer’s test (mm)	*r* = −0.14	*r* = − 0.18
	*P* = 0.27	*P* = 0.43
TBUT (s)	*r* = 0.12	*r* = − 0.40
	*P* = 0.67	*P* = 0.20
Corneal fluorescein staining score	*r* = 0.28	*r* = 0.39
	*P* = 0.48	*P* = 0.03^a^
Lid margin abnormality score	*r* = 0.30	*r* = 0.16
	*P* = 0.18	*P* = 0.51

Spearman’s correlation test was used

*Meiboscore (U + L)* meiboscores of upper eyelid and lower eyelid at post-RT 6 months were added up; *OAML* ocular adnexal mucosa-associated lymphoid tissue lymphoma, *OSDI* ocular surface disease index, *TBUT* tear film break-up time

^a^Values with statistical significance are indicated with an asterisk (*)

The symptoms of ocular dryness were at their most severe right after RT in both the conjunctival lymphoma group and the “orbital-type” lymphoma group (OSDI score; 29.5 ± 23.5 and 39.2 ± 28.3 at 1 month post-RT, respectively) but a gradual resolution of dry eye was noted in most patients with conjunctival lymphoma with the application of artificial tears and lubricating eye drops, whereas symptoms remained persistent in patients with “orbital-type” lymphoma, as shown in Fig. 5 by OSDI score. Patients in whom the increase in meiboscore was less than 1 at 6 months post-RT demonstrated an average OSDI score increase of 2.5. On the other hand, those with an increase in their meiboscore of more than 1 had an average OSDI score increase of 19.4. This difference showed statistical significance (*P* < 0.001), indicating that with minimal or absent morphological changes in the meibomian glands, dry eye symptoms were also either entirely absent or only mild in nature (i.e., the patient demonstrated occasional mild tearing, foreign body sensation without a reduction in visual acuity, and easy improvement with medical treatment).

No patient had development of severe dry eye with corneal erosion or vision compromise. Severe cases requiring autologous serum, punctal plugs, or therapeutic contact lenses were not present. The only observed corneal reactions were superficial punctate keratopathy, affecting 13 (29.5%) eyes in the conjunctival group and 14 (70%) eyes in the “orbital-type” group.

## Discussion

This study investigated the adverse effects of the EBRT on the meibomian glands in patients with OAML. The EBRT induced changes in their morphological structures and concomitantly gave rise to dry eye syndrome. In the current study, these adverse effects seemed to be irreversible during the 6 months follow-up period. Generally, it is thought that the gland tissue cannot be recovered once destroyed, but further study with longer follow-up period is required to reveal the reversibility of the meibomian gland damage after radiation.

Radiotherapy techniques typically varied according to the disease location in the orbit. Tumors confined to superficial structures such as the conjunctiva were usually approached with a direct electron beam, whereas orbital tumors were treated with X-ray or photon beams [16–18]. The electron beam is effective in managing superficial lesions yet does not penetrate to deeper tissues, thereby having a relatively protective effect on the inside structures. On the other hand, while X-rays or photon beams were used to treat “orbital-type” lymphomas as they can penetrate deeper, this inevitably irradiates the adnexal ocular structures. The eyes that received RT at a higher radiation dose and energy level or that received X-rays rather than electron energy were more vulnerable to dry eye syndrome, making patients with “orbital-type” lymphoma more susceptible because of the deeper location and relatively large size of the tumor.

In this study, the morphological changes in the meibomian glands occurred in early period after RT. In most cases, the irradiated eye did not progress over time, though some patients did demonstrate progressive changes. The probability of damage occurring in the meibomian glands and the development of MGD or dry eye were dependent on the radiation dose and, possibly on energy type. Post-radiation dry eye syndrome was more likely to occur in cases of EBRT with higher radiation doses and energy levels. Moreover, the use of X-rays seemed to predispose irradiated eyes to being more vulnerable to dry eye syndrome. The post-radiation meibomian gland injury was more notably found in patients with “orbital-type” lymphoma. Taken altogether, the patients with conjunctival lymphoma had better post-radiation prognoses than did those with “orbital-type” lymphoma with respect to meibomian gland injury, tear film instability and dry eye syndrome.

Normal meibomian glands have the appearance of grape-like clusters with visible saccular acini that are arranged perpendicular to the lid margin [22]. Abnormal meibomian glands often show dilated ducts and tortuous glands, which eventually results in gland dropout or shortening. These features are consistent with previous histopathologic study of exenteration specimens that were irradiated due to malignant tumors. In the study by Karp et al. [23], as sequelae of irradiation, acinar atrophy and cystically dilated meibomian ducts were seen along with squamous metaplasia of the meibomian glands. They reported that histopathologic findings seemed to indicate that the meibomian glands, which are sebaceous in nature, are more sensitive to irradiation and are more permanently altered than are the sebaceous glands of Zeis. In the present study, ducts and acini seemed to be constricted as slender ducts were seen in meibography in early period of RT. It is believed that obstructive type of meibomian gland dysfunction is the most commonly found type, with cornification of the lid margins and dilatation of ducts. However, dilatation of ducts was not found in this study. Possible pathogenesis of post-RT meibomian gland dysfunction may be the direct damage from irradiation which could induce acinar cell dysfunction and atrophy of the gland tissue and this would eventually result in the loss of the gland.

Among patients with no significant injury to the meibomian glands, most did not develop dry eye or only had mild dry eye symptoms. However, with acknowledgeable structural damage to the meibomian glands, ocular dryness was persistently symptomatic even after the subsidence of the acute radiation reaction. With considerable loss of gland, conventional treatment, such as warm compression and massage of lid was ineffective. Instead, such patients were treated by topical lubricants such as replacement of the lipid layer.

Three patients with lacrimal gland lymphoma had more pronounced dry eye symptoms. There is not yet any study that has aimed to identify whether the cause of post-radiation dry eye is more attributable to meibomian gland dysfunction or to lacrimal apparatus atrophy in the case of lacrimal gland tumor. However, this study clearly suggests that when both the meibomian glands and lacrimal gland apparatus are damaged by RT, severe dry eye syndrome is unavoidable.

It is reported that RT planning using IMRT reduces the dose administered to the surrounding structures and may help to minimize the risks of RT-induced dry eye [24]. Surprisingly, meibomian gland damages were not found in the eyes that received IMRT with the radiation beam target being the mass only. These patients had less dry eye symptoms than did the patients who received conventional 3D-RT with the beam directed on the entire orbital socket. However, further study with a large number of such cases is needed for statistical analysis.

The limitation of the current study is that the two groups had a different number of patients. The “orbital-type” group had a small number of subjects compared to the conjunctival group because most patients with conjunctival lymphoma had primary radiotherapy whereas many of those with “orbital-type” lymphoma had chemotherapy rather than radiotherapy according to physician’s clinical judgement.

## Conclusions

The present study tried to differentiate the radiation effects in two groups of patients according to the location of OAML as higher radiation energy and dose are required in deeply placed tumors or large tumors. This new approach provides evidence that after RT, more severe dry eye is inducible by the presence of more morphological damage in the meibomian glands in “orbital-type” lymphoma. Thus, we carefully suggest that clinicians should be cautioned, as many “orbital-type” lymphoma patients will have some degree of injury to the meibomian glands and that those patients should be well informed of post-RT persistent dry eye which may not be easily curable. An examination of the meibomian glands should be especially considered in patients with “orbital-type” lymphoma having post-RT dry eye.

## Data Availability

The datasets used and analyzed during the current study are available from the corresponding author on reasonable request.

## Abbreviations

cGy: centigray
MeV: Megaelectron-volt
MV: Megavolt
OAML: Ocular adnexal mucosa-associated lymphoid tissue lymphoma
OSDI: Ocular Surface Disease Index
RT: Radiation therapy
TBUT: Tear film break-up time

## References

[1] Stefanovic A, Lossos IS (2009). Extranodal marginal zone lymphoma of the ocular adnexa. Blood.

[2] Bhatia S, Paulino AC, Buatti JM, Mayr NA, Wen BC (2002). Curative radiotherapy for primary orbital lymphoma. Int J Radiat Oncol Biol Phys.

[3] Galieni P, Polito E, Leccisotti A, Marotta G, Lasi S, Bigazzi C (1997). Localized orbital lymphoma. Haematologica.

[4] Hasegawa M, Kojima M, Shioya M, Tamaki Y, Saitoh JI, Sakurai H (2003). Treatment results of radiotherapy for malignant lymphoma of the orbit and histopathologic review according to the WHO classification. Int J Radiat Oncol Biol Phys.

[5] Sasai K, Yamabe H, Dodo Y, Kashii S, Nagata Y, Hiraoka M (2001). Non-Hodgkin’s lymphoma of the ocular adnexa. Acta Oncol.

[6] Jeganathan VS, Wirth A, MacManus MP (2011). Ocular risks from orbital and periorbital radiation therapy: a critical review. Int J Radiat Oncol Biol Phys.

[7] Kennerdell JS, Flores NE, Hartsock RJ (1999). Low-dose radiotherapy for lymphoid lesions of the orbit and ocular adnexa. Ophthal Plast Reconstr Surg.

[8] Parsons JT, Bova FJ, Fitzgerald CR, Mendenhall WM, Million RR (1994). Severe dry-eye syndrome following external beam irradiation. Int J Radiat Oncol Biol Phys.

[9] Parsons JT, Bova FJ, Fitzgerald CR, Mendenhall WM, Million RR (1994). Radiation optic neuropathy after megavoltage external-beam irradiation: analysis of time-dose factors. Int J Radiat Oncol Biol Phys.

[10] Merriam GR, Focht EF (1957). A clinical study of radiation cataracts and the relationship to dose. Am J Roentgenol Radium Therapy, Nucl Med.

[11] Egbert PR, Donaldson SS, Moazed K, Rosenthal AR (1978). Visual results and ocular complications following radiotherapy for retinoblastoma. Arch Ophthalmol.

[12] Parsons JT, Fitzgerald CR, Hood CI, Ellingwood KE, Bova FJ, Million RR (1983). The effects of irradiation on the eye and optic nerve. Int J Radiat Oncol Biol Phys.

[13] Chan RC, Shukovsky LJ (1976). Effects of irradiation on the eye. Radiology.

[14] Chhadva P, Goldhardt R, Galor A (2017). Meibomian gland disease: the role of gland dysfunction in dry eye disease. Ophthalmology.

[15] Nelson JD, Shimazaki J, Benitez-del-Castillo JM, Craig JP, McCulley JP, Den S (2011). The international workshop on meibomian gland dysfunction: report of the definition and classification subcommittee. Invest Ophthalmol Vis Sci.

[16] Letschert JG, Gonzalez Gonzalez D, Oskam J, Koornneef L, van Dijk JD, Boukes R (1991). Results of radiotherapy in patients with stage I orbital non-Hodgkin's lymphoma. Radiother Oncol.

[17] Bessell EM, Henk JM, Whitelocke RA, Wright JE (1987). Ocular morbidity after radiotherapy of orbital and conjunctival lymphoma. Eye (Lond).

[18] Jereb B, Lee H, Jakobiec FA, Kutcher J (1984). Radiation therapy of conjunctival and orbital lymphoid tumors. Int J Radiat Oncol Biol Phys.

[19] Hwang HS, Park CW, Joo CK (2013). Novel noncontact meibography with anterior segment optical coherence tomography: Hosik meibography. Cornea.

[20] Arita R, Itoh K, Maeda S, Maeda K, Furuta A, Fukuoka S (2009). Proposed diagnostic criteria for obstructive meibomian gland dysfunction. Ophthalmology.

[21] Schiffman RM, Christianson MD, Jacobsen G, Hirsch JD, Reis BL (2000). Reliability and validity of the ocular surface disease index. Arch Ophthalmol.

[22] Sirigu P, Shen RL, Pinto da Silva P (1992). Human meibomian glands: the ultrastructure of acinar cells as viewed by thin section and freeze-fracture transmission electron microscopies. Invest Ophthalmol Vis Sci.

[23] Karp LA, Streeten BW, Cogan DG (1979). Radiation-induced atrophy of the Meibomian gland. Arch Ophthalmol.

[24] Goyal S, Cohler A, Camporeale J, Narra V, Yue NJ (2008). Intensity-modulated radiation therapy for orbital lymphoma. Radiat Med.

